# MomBod: Exploring body-related perceptions and the influence of physical activity imagery among postpartum women

**DOI:** 10.1177/13591053261419659

**Published:** 2026-02-22

**Authors:** Iris A. Lesser, Megan Filiatrault, Amanda Wurz, Taniya S. Nagpal

**Affiliations:** 1 University of the Fraser Valley, Chilliwack, BC, Canada; 2 University of Calgary, AB, Canada; 3 Sport and Recreation, Edmonton, AB, Canada

**Keywords:** postpartum, physical activity, body, imagery, exercise

## Abstract

Despite the known benefits of PA, many women do not engage in sufficient PA. This study explored postpartum women’s perceptions of their body and PA and evaluated whether “realistic” PA imagery may influence perspectives around PA. We created imagery portraying safe, appropriate postpartum exercises and conducted an explanatory sequential mixed methods study. Given the influence of postpartum weight retention on body dissatisfaction we further explored experiences based on postpartum weight retention. Participants (*n* = 33, *M* = 31 years) <12 months postpartum completed a questionnaire, and *n* = 21 completed semi-structured interviews. Quantitative data were analyzed with descriptive statistics, qualitative data with reflexive thematic analysis. No significant differences were found in body dissatisfaction and PA between weight loss and weight retention groups. Three themes emerged: (1) perceptions of body image and size postpartum, (2) PA imagery preferences, (3) sources of PA information. Findings suggest women experience body dissatisfaction after viewing imagery and desire “realistic” PA imagery.

## Introduction

There is a societal expectation that motherhood is marked by experiences of happiness and joy. However, the experience is significantly more nuanced than that – with many women experiencing physical and psychological challenges, leading to incongruence between expectations and reality (Yelland et al., 2010). Physical activity (PA) is one strategy associated with a range of positive physical and psychological health outcomes that has been shown to be beneficial for new mothers (Davenport et al., 2025; Dipietro et al., 2019; Lesser et al., 2023); including supporting self-compassion (Lesser et al., 2024), decreasing anxiety (Hatfield et al., 2022), and improving fatigue (Davenport et al., 2025). Despite the known benefits of PA, many women do not engage in sufficient PA to achieve positive physical and psychological health outcomes postpartum (Chu et al., 2023; Lesser et al., 2023).

There are likely many reasons for reduced PA postpartum including lack of support, time, and motivation, feelings of fatigue (Saligheh et al., 2016), lack of education around how to return to PA (Ritondo et al., 2024), and challenges obtaining medical clearance (Davenport et al., 2025). Moreover, after the birth of a child, women are faced with (often) rapidly changing body weight, shape, size, and function (Clark et al., 2009a), which can further negatively impact PA. For some, lower and maladaptive PA patterns have been observed, wherein some women report exercising to avoid feeling guilty (Peralta et al., 2021; Raspovic et al., 2022; Thompson and Bardone-Cone, 2022). In other cases, lower and/or maladaptive PA has been suggested to be influenced by one’s body shape and size with those of higher body mass showing higher body dissatisfaction and engaging in more PA (Tornero-Quiñones et al., 2023).

While it is perhaps unsurprising that body shape and size can adversely influence PA and experience (Thompson and Bardone-Cone, 2022), postpartum may represent a time when women struggle more with not only PA engagement but body dissatisfaction. While some women adjust to their changed body postpartum, others struggle to adapt, not only engaging in less PA, but experiencing greater dissatisfaction with appearance (Spinoni et al., 2023). Over 50% of women have reported experiencing body dissatisfaction postpartum (Vanderkruik et al., 2022). Notably, women who report higher levels of self-compassion (defined as kindness toward oneself; Neff, 2003) after the birth of a child have been found to have greater levels of body appreciation and lower levels of depression (Rosenbaum et al., 2024) which could conceivably also result in lower levels of body dissatisfaction. Furthermore, body dissatisfaction is typically lower among those who retain postpartum weight (Chen and Chang, 2023; Gjerdingen et al., 2009; Huang and Dai, 2007). Those with postpartum weight retention are more likely to desire weight loss (Huang and Dai, 2007) and PA engagement may therefore diverge between those who maintain postpartum weight retention and those who return to their pre-pregnancy weight.

Within Western cultures, the “bounce-back” narrative – the expectation that postpartum weight will be lost quickly, and the body will return to its pre-pregnancy state (Clark et al., 2009b) – dominates and is associated with greater body dissatisfaction (Horton et al., 2025). This issue may be further compounded by comparison – particularly via social media. Postpartum women have reported using social media to access health information, including PA guidance (Baker and Yang, 2018; Nagl et al., 2021) and that social media was specifically associated with the pressure to “bounce-back” to their pre-pregnant state (Nagl et al., 2021). Unsurprisingly, the more women engage with social media, the greater their body dissatisfaction (Tang et al., 2022) particularly if they had a higher social comparison orientation (Wenhold et al., 2025). Further, it seems that in the tumultuous postpartum period, reconciling one’s “new” body while engaging in comparison via social media may not only lead to greater body dissatisfaction, but also result in problematic PA (Tang et al., 2022) or limit/reduce PA (Raspovic et al., 2022).

As a first step to better support postpartum women, we created more “realistic” PA content that sought to move beyond the dominant Western ideal and images commonly portrayed (e.g. White, slim, and fit women; (Jones et al., 2024; Tiggemann and Zaccardo, 2018). The PA imagery was created by the research team over 1 month and included models who were less than 12 weeks postpartum and volunteered to participate as models to create realistic imagery of postpartum women completing common postpartum rehabilitation exercises. Exercises were chosen with expertise from a physiotherapist with certification in pelvic female health to be appropriate postpartum after vaginal or surgical birth (Supplemental File 1).

The overarching purposes of this study was to explore postpartum women’s perceptions of their body (body dissatisfaction), PA, and related constructs (PA identity, exercise self-efficacy, self-compassion) and evaluate whether our newly created PA imagery comprised of more “realistic” and diverse bodies influenced these perceptions. Specific objectives included: (1) describing body dissatisfaction, PA, and related constructs (PA identity, exercise self-efficacy, self-compassion) among postpartum women; (2) exploring postpartum women’s perspectives on our newly created PA imagery (as compared to stock imagery of models portraying the same image); and (3) as an exploratory objective, explore whether there are differences in scores or experiences based on weight status (i.e. women who have lost vs retained postpartum weight).

## Methods

An explanatory sequential mixed method was conducted, wherein first quantitative data (i.e. surveys) were collected, and then qualitative data (i.e. interviews) were collected. Quantitative and qualitative data were then integrated, compared, and knowledge accrued through both means was used to extend understandings. The University of the Fraser Valley Human Ethics Research Board approved this study (HREB #101584) and all participants provided online informed consent through Survey Monkey.

### Recruitment and study population

Potential participants were recruited through personal and university social media as well as word-of-mouth between August 2024 and January 2025. Participants were eligible to participate in the study if they were less than 12 months postpartum and were able to understand and speak English. The subset who completed interviews were provided with a $25 gift card to compensate for their time.

### Procedures

**Figure 1.**
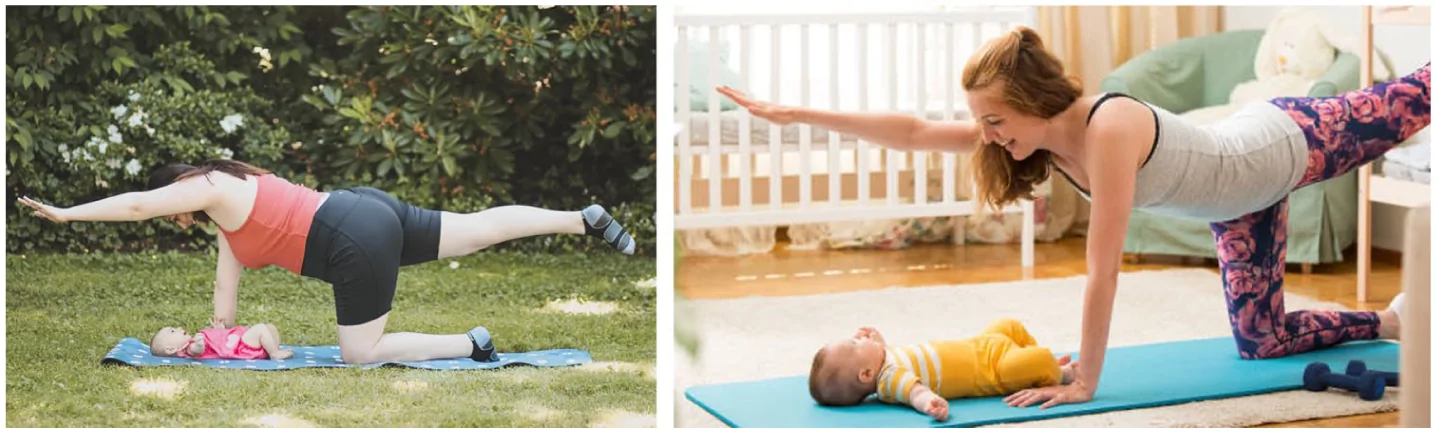
Newly created imagery of a postpartum mother completing bird-dog exercise compared to a stock image found under the search term “postpartum exercise” of a bird-dog exercise.

**Figure 2.**
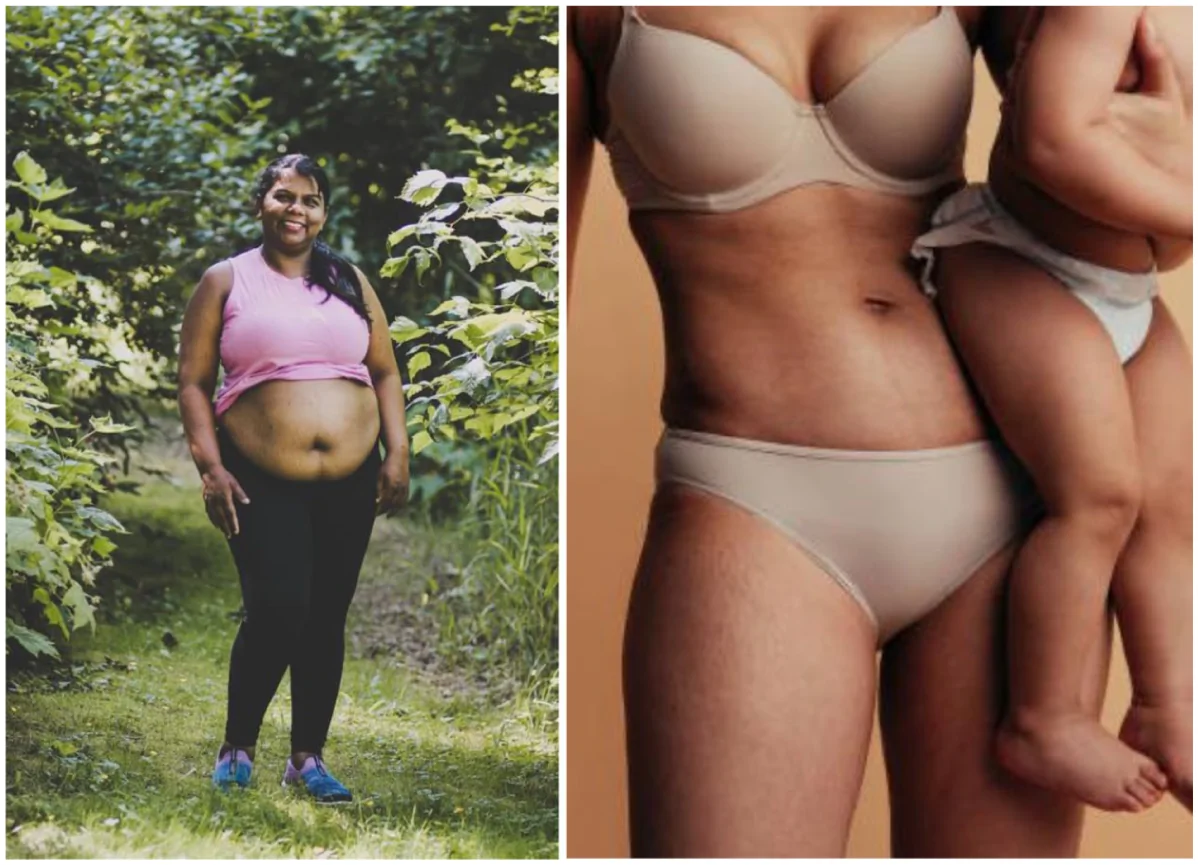
Newly created imagery of a postpartum stomach compared to a stock image found under the search term “postpartum belly.”

Participants completed an online survey which included: (1) demographic and maternal health information, (2) PA participation and (3) exercise self-efficacy, PA identity, self-compassion, and body dissatisfaction. See Measures below. Following the survey, a subset of purposefully recruited participants (postpartum weight retention and postpartum weight loss based on participants self-reporting returning to pre-pregnancy weight or not) completed a semi-structured interview (see Semi-Structured Interview for more details, below). Interviews were conducted at a time and via a mode (e.g. online, in-person) of participants’ choosing. First, participants were asked questions exploring perceptions of their body postpartum, PA information access and PA imagery. Participants were then presented with a PowerPoint slideshow with stock imagery and our newly created exercise imagery and were asked questions related to how the different images (mom A vs mom B) made them feel about their bodies and PA engagement. Of note, for each of the rehabilitation exercises in our newly created imagery, we matched as closely as possible with a stock photo of a mother engaging in a similar exercise using the search term “postpartum exercise.” Comparisons of our newly created imagery (without exercises, i.e. women showing their postpartum stomach) to stock photos of a postpartum stomach were also shown (see Supplemental File 1 and sample comparisons are shown in Figures 1 and 2, below). The order in which images were shown were reversed (stock imagery and then newly created imagery or vice versa) for every other participant to minimize bias in the order of representation. A total of 14 exercises and 3 non-exercise comparisons were displayed, and each image was shown for 5 seconds.

#### Survey measures

##### Demographic and maternal health

Participants were asked their age, their ethnicity, how many children they had and the current age of their youngest child. Participants additionally reported their gestational weight gain from their most recent pregnancy and whether they had postpartum weight retention described as being heavier than pre-pregnancy.

##### PA

Participants were asked to report whether they were meeting the PA guidelines postpartum: “In the postpartum period (since you have been cleared for PA after the birth of your child), would you describe yourself as physically active (>150 minutes of moderate to vigorous effort PA such as brisk walking per week)?” (Davenport et al., 2020).

##### Exercise self-efficacy

Participant’s self-efficacy was assessed using the exercise self-efficacy scale (Neupert et al., 2009). This nine-item scale is measured on a 4-point scale (1 = very *sure* to 4 = not *at all sure*) and uses the stem: “How sure are you that you will do each of the following. . .” (e.g. “. . .exercise when tired”). A higher score equates to greater exercise self-efficacy. The internal consistency reliability was found to be good in our previous work with postpartum women (α = 0.88, 95% CI [0.86, 0.90]; Lesser et al., 2023).

##### Self-compassion

Participant’s self-compassion was assessed using the self-compassion short-form scale (Raes et al., 2011). This 12-item scale is measured on a 5-point scale (1 = almost *never* to 5 = almost *always*; e.g. “When something upsets me, I try to keep my emotions in balance”). Higher scores indicate greater self-compassion. The internal consistency reliability was found to be good in our previous work with postpartum women (α = 0.84, 95% CI [0.81, 0.86] (Lesser et al., 2023).

##### Physical activity identity measurement scale

Participant’s identification with their athletic role was assessed using the Athlete Identity Measurement Scale (AIMS; Brewer et al., 1993). This seven-item scale is measured on a 5-point scale (1 = strongly *disagree* to 5 = strongly *agree*; e.g. “I consider myself an athlete”). As we were interested in PA identity rather than athletic identity, changes were made to the sport items to reflect PA (e.g. “I have many goals related to sport” was changed to “I have many goals related to PA”). Higher scores indicate a greater PA identity. The internal consistency reliability for this scale was found to be acceptable in our work with postpartum women (α = 0.73, 95% CI [0.65, 0.75]; Lesser et al., 2023).

##### Body dissatisfaction

Participant body dissatisfaction was assessed using the Body Shape Questionnaire (Evans and Dolan, 1993). This eight-item scale is measured on a 6-point scale (1 = never to 6 = always; e.g. “Have you been worried about your flesh not being firm enough?”). A higher score indicates greater body dissatisfaction. The internal consistency reliability for this scale was found to be excellent in our work with postpartum women (α = 0.90, 95% CI [0.88, 0.91]; Lesser et al., 2023).

##### Qualitative data collection

Semi-structured, one-on-one interviews were conducted by a female research assistant (A2) who supported the newly created imagery and oversaw elements of the study conduct including recruitment, informed consent and survey completion. Interviews followed a guide and were audio-recorded and transcribed verbatim. As above, questions asked participants to share their experiences with PA, their body dissatisfaction, where they access PA information and after being presented with the slideshow of PA imagery, whether imagery impacted PA confidence postpartum.

### Data analysis

Quantitative data covering body dissatisfaction, PA participation and related constructs (exercise self-efficacy, PA identity, self-compassion) were analyzed using descriptive statistics in SPSS Version 29.0 to address objective 1. As well, to address objective 3, differences between those who reported postpartum weight loss and those who reported postpartum weight retention were evaluated using independent *t*-tests (continuous data) and Chi square analyses (ordinal data) with alpha set to 0.05.

Qualitative data were analyzed following guidance for reflective thematic analysis (Braun and Clarke, 2019, 2025) to address objectives 2 and 3. First, data were transcribed verbatim. Next, the first author (A1) familiarized themselves with the data through re-reading the transcription to gain an understanding of the data. This analysis was then reviewed by A2 who conducted the interviews. A1 and A2 then participated in an iterative process of refining themes and subthemes to co-produce Table 2 and shared with A3 and A4 who reviewed the analysis and provided critical commentary. Multiple iterations ensued wherein raw data was re-visited, and interpretations were challenged. As a final step, themes were re-reviewed by all authors to ensure alignment. To improve the ease of reading, filler words (such as like, uhm) were removed and pseudonyms were used to preserve anonymity.

## Results

Quantitative descriptive data are presented below followed by outcomes from qualitative thematic analysis. Over 3 months, 33 women provided informed consent and participated in the online survey. The average age of participants was 31 (4.8) years. For most participants this was their first (*n* = 21, 64%) or second (*n* = 9, 27%) child. Most participants were of Black or White ethnicity (*n* = 16, 48% and *n* = 14, 43% respectively). On average, women reported gestational weight gain of 38.0 lbs (22.2). At the time of survey completion their youngest child was 24.2 (13.0) weeks of age. All demographic summary data are presented in Table 1. When comparing postpartum weight loss versus postpartum weight retention there were no significant differences in maternal age, number of children, gestational weight gain, ethnicity, or whether participants reported meeting PA guidelines (*p* > 0.05; Table 1). However, those who reported lost postpartum weight had an older child than those who retained postpartum weight (31 weeks vs 19 weeks, *p* < 0.05; Table 1).

**Table 1.** Maternal demographics among those who reported postpartum weight loss versus those who reported postpartum weight retention. Continuous data assessed using an independent *t*-test (means and standard deviation), ordinal data (frequency and percentile) using a Chi square analysis.

Maternal Demographics	All participants (*n* = 33)	Postpartum weight loss (*n* = 15)	Postpartum weight retention (*n* = 18)	*p*-Value
Maternal age	31.0 (4.8)	31.9 (5.5)	30.3 (4.0)	0.437
First child	21 (64%)	9 (60.0%)	12 (67.0%)	0.980
Gestational weight gain (lbs)	38.0 (22.2)	30.2 (13.7)	44.2 (25.8)	0.076
Age of child (weeks)	24.2 (13.0)	30.7 (11.4)	18.8 (11.9)*	0.007
Meeting PA guidelines	23 (70%)	11 (79%)	12 (67%)	0.457

Note: *denotes statisticaly singificant at *p* < 0.05

With regards to describing body dissatisfaction, PA, and related constructs among postpartum women, 70% of women reported meeting PA guidelines of 150 minutes of moderate to vigorous PA per week. Participants had an average score of 24.0 (7.9) for body dissatisfaction out of a possible score of 48 (most body dissatisfaction; see Table 2 for full set of descriptive statistics). With regards to objective 3, there were no significant differences between those who lost postpartum weight and those who retained postpartum weight in measures of exercise self-efficacy, PA identity, body dissatisfaction or self-compassion (Table 2). Of note, these patterns (i.e. no significant differences in constructs) were observed when comparing participants who did and did not retain weight and who completed an interview.

**Table 2.** Questionnaire scores among those who reported postpartum weight loss versus those who reported postpartum weight retention. Continuous data were compared using an independent *t*-test.

Measures	All participants (*n* = 33)	Postpartum weight loss (*n* = 15)	Postpartum weight retention (*n* = 18)	*p*-Value
Exercise self-efficacy	23.3 (6.7)	22.6 (7.6)	23.8 (6.1)	0.642
PA identity	33.3 (8.2)	34.0 (7.5)	32.8 (8.9)	0.696
Body dissatisfaction	24.0 (7.9)	24.9 (7.4)	23.3 (8.5)	0.584
Self-compassion	61.3 (11.4)	60.8 (9.71)	61.7 (12.9)	0.822

Despite no changes in quantitative data between those who reported postpartum weight loss and those who reported postpartum weight retention, there were obvious differences in women’s accounts of body dissatisfaction and social comparison based on weight status. Indeed, findings from qualitative data suggest notable differences between those who lost postpartum weight versus retained postpartum weight in a select number of themes (see Supplemental File 2). Reflective thematic analysis revealed three themes: (1) perceptions of body image and size postpartum, (2) PA imagery preferences, and needs (3) sources of PA information.

### Perception around body image and size postpartum

This theme captures when participants spoke about how they felt about their body and size since giving birth to their child. A notable difference was evident between those who retained versus lost postpartum weight wherein participants who reported postpartum weight retention saw their bodies as needing to be fixed. These women discussed a mix of wanting to be proud of what their body had achieved but not always liking what they saw in the mirror. Those who reported postpartum weight loss on the other hand embraced their body changes as scars of motherhood, while these women still reported having days where they felt less comfortable in their skin, they were overall happy with their changed shape and were able to give themselves grace. Latent analysis revealed flip flopping in the responses of postpartum women around the notion of body appreciation with the interviewer (A2), discerning that participants often appeared to be trying to find the “right answer” when it came to discussing postpartum body image, but that other answers denoted a subconscious pursuit of an idealized body.

#### The body as a battleground

This subtheme captures when women who had retained postpartum weight discussed a desire to embrace their changed body given the feat it had accomplished in housing and birthing their child. However, these positive comments often ended with an expression of body dissatisfaction. “*I think my body’s amazing for what it did for getting pregnant and having babies. But it is hard because your body changes so much.”* (Alyssa, postpartum weight retention). Similarly, Leah (postpartum weight loss) shared *“You don’t feel like your body belongs to you anymore,”* highlighting a sense of disconnection from the postpartum body. Participants expressed wanting to embrace the motherhood experience and not dwelling on their physical experience but expressing discontent. Hannah (postpartum weight retention) states: *“I’m definitely trying to change the narrative, but it’s so hard. Like, I can look in the mirror and be like, I’m happy with who I am. . . .and then I look at pictures. . .”* This may be particularly challenging for her as she describes how her job in health and fitness made acceptance more challenging *“it’s hard to disconnect that like my body doesn’t necessarily indicate my health status.”* There was one notable exception in Kayla who had experienced postpartum weight loss (postpartum weight loss) but struggled with her appearance *“I’m also like, I’m also only six months postpartum, so I take that with a grain of salt, but it definitely looks different, and I am struggling with it.”*

#### Embracing the “mom-bod”

This subtheme captures perspectives from those who lost postpartum weight who described embracing their motherhood scars, loose belly and stretch marks more so than those who retained postpartum weight allowing these women to forego the bounce back narrative to appear untouched by motherhood. Jasmine (postpartum weight loss) states: *“I don’t feel the necessary pressure to bounce back to my normal body. And I’ve also sort of gained some scars and so I’m embracing them. . . I’m filled with joy just to look the way I look and to be who I am, to be a mother to someone.”* This shift in perspective was supported by Kayla (postpartum weight loss) who noted a change in mindset: *“Postpartum, I feel like I’m a little bit more accepting. I give myself more grace.”* For some, motherhood helped them embrace their bodies in a way they couldn’t before: *“I’m also just in awe. Like my skin is different; it’s a different texture. It feels different than it did before, like it’s very loose, you know? So, it looks different, but I’m just really proud of it. . .”* Dana (postpartum weight loss). Likewise, Leah (postpartum weight loss) stated, *“if anything, I just have more appreciation for my body because of the incredible miracle that it produced.”*

### PA imagery preferences and needs

This theme captures the importance of having postpartum PA imagery reflective of diverse body shapes and size. Of note, most of the women who retained postpartum weight found that the diverse imagery made them feel more confident. Regardless of weight status, women agreed that the exercise being shown was more important to their confidence around PA engagement than the size of the individual.

#### Retention of postpartum weight impacts how women view exercise imagery

This subtheme captures perspectives of participants who lost postpartum weight being more likely to focus on the esthetics of the image; whether the mom was wearing nice workout clothes, the background of the image being too esthetic and whether the baby appeared to be happy. *“It would be the mix of in the house, outside, in a hotel gym, in an unaesthetic basement gym. . . where they’re doing it, and without the laundry being clean first, you know”* (Dana, postpartum weight loss). For Elizabeth (postpartum weight loss) the importance of the diverse imagery was more in relation to the appearance of the mother appreciating the need for imagery where: *“women may look like if they rolled out of bed and wanted to squeeze a 20-minute home workout in.”* Mary (postpartum weight loss) specifically focused on the baby in the stock imagery being happy and content *“babies are different than others, so they might be dealing with a very colicky, crying baby or something, and that could affect them in a certain way.”* However, this wasn’t just unique to mothers who lost postpartum weight as Laura who retained postpartum weight describes *“it would be nice to see imagery of moms trying to do a class and how. . . getting interrupted because their kid is doing something like, how do they recover when everybody’s not . . . sitting perfectly in a row and the babies are all just happy and sitting there.”*

Mary who retained postpartum weight felt that she related more to the stock imagery but that seeing our more diverse imagery boosted her confidence around PA: *“My muscles don’t do what they used to, not as strong as I was before. So, it’s encouraging seeing the ‘diverse image’ exercises, because they’re all like getting into it where they’re at. You know what I mean, whereas the other photos obviously like I said look a bit more staged and glamorized and not as real and relatable.”* Mothers discussed noticing the differences in the challenges of the exercises and how the stock imagery included exercises that they thought were too challenging and therefore they were more confident in doing the exercises from the realistic imagery. This was true whether mothers lost postpartum weight or retained it *“mom one did more yoga based simpler movements that are designed by a pelvic health physio for postpartum, and mom two is doing exercises that are a little bit too advanced for the newly postpartum woman”* (Anna, postpartum weight loss). This was explained by Selena who retained postpartum weight as “*I prefer the bigger mom, because she’s doing the soft exercises.”* Those who retained postpartum weight were more likely to focus on the body size, often stating they related more to the diverse imagery “*it’s so realistic. It’s how everyone looks after pregnancy. I mean, ‘stock imagery’ really looks like they just picked up a model who hasn’t been pregnant before and had to do a shoot or something. . . definitely feel more motivated to work out because she’s doing it.”* (Julie, postpartum weight retention).

However, for some mothers who struggled with social comparison, they were still more motivated by the stock imagery and preferred it as it was where they wanted to be in terms of body size and fitness. Hannah who retained postpartum weight shared that her initial reaction was *“I still kind of wish I looked more like ‘stock imagery’,”* however, she also states: *“it’s reassuring to know that there’s that I’m definitely not the only mother* that is *going through this stage of life that doesn’t look like the Pilates mom in there*. . .″Overall, mothers regardless of body size or weight retention felt that it was important to have a diverse set of images that were representative of many shapes, size and ethnicities. The importance of racial diversity and size diversity was highlighted by Nicole (postpartum weight retention) who wanted to see herself in imagery *“like, plus size, a little bit of plus size, then also blacks”*

#### Social comparison hinders confidence and self-acceptance in motherhood

This subtheme captures how comparing to other impacts body satisfaction postpartum. When viewing other postpartum imagery, women were more likely to discuss not fitting into the societal ideal and that they felt behind in their own PA and body image journey. Hannah who retained postpartum weight discusses how social comparison led her to being more negative about her own challenges *“I have mom friends that didn’t gain much weight at all in pregnancy, and they look very similar [. . .] postpartum as they did pre pregnancy. And that’s when comparisons start to happen, and it starts to get a little bit more negative self-talk going on there.”* This same comparison is noted by Julie who retained postpartum weight: *“I felt so insecure, you know, most of the time, because I know I’ve seen so many women have children, but they bounced back to shape so easily. So, I kind of wonder, why is it so hard for me? Why can’t I achieve that?”* Ashley who retained postpartum weight discusses how seeing the stock images next to the realistic imagery made it obvious that one is obviously a model but that when viewing images outside of this context she struggles with recognizing that comparison *“And that’s when the comparisons against yourself happen* versus *what a wide variety of other people in your position look like.”* She describes how you suddenly see someone postpartum who is already fit and how “*it’s like you were pregnant with abs.”* This is noted by Sarah (postpartum weight retention) who discusses the discouragement which takes place when you don’t recognize yourself in other mothers “. . .*made me feel like my journey isn’t as valid or that I’m behind in some way.”*

Julie (postpartum weight retention), who is also challenged with how she feels about her weight and size, discusses mostly relying on her physician for health information but that when she engages with social media she notes the social comparison *“it’s not always the same with what’s in reality, because everybody’s trying to portray a perfect body, a perfect, you know size, but I’ve learned to not listen to social media as much.”* After viewing “realistic” imagery Julie states *“now I feel like I’m not alone, and it’s more normal. It’s harder for us.”* However, there are solutions to getting caught up in these comparisons with Laura (postpartum weight retention) stating that there is common humanity if you find the right people: *“it’s also possible to create a worldview that does support you. . .find your group.”*

#### Social comparison supports common humanity in motherhood

This subtheme captures positive perspectives of social comparison wherein looking to others allowed for common humanity. Taylor who retained postpartum weight states that *“we’ve got different body shapes. We’ve got different body sizes. We’ve got people with different lived experiences. I enjoy reading from the lived experiences of pregnant moms and women we just give birth, and this has kind of prepared me for this phase, for this journey knowing it’s not always going to be perfect.”* Alyssa (postpartum weight retention) discusses how when she sees other postpartum women exercising that are smaller than her, she just recognizes them as having a smaller body size, and it doesn’t make her uncomfortable. Emily who retained postpartum weight further recognizes the limits of social comparison *“you cannot replicate someone elses imagery. You just have to be true to yourself.”*

### Sources of PA information

This theme captures where postpartum women sought out PA information postpartum. Most mothers discussed using credible sources when accessing PA information postpartum. Reliance on health care providers, pelvic physiotherapists or specialized programing for postpartum women were all cited as being the most common avenue for accessing information. Programing that was done by fitness instructors who self-described being mothers was preferred by most. While some struggled with social comparison, most mothers embraced having a fitness instructor who was slim and fit as they felt it was something that they wanted to achieve. One mother, who had a background in fitness and modeling and had since struggled with body image and weight gain, discussed feeling as though postpartum PA information wasn’t catered to her as it was geared to individuals struggling with obesity or who were concerned about movement after surgical birth. Another mother seconded this, feeling as though there was a gap between what was discussed in videos targeted to postpartum exercise and what she was looking to do. This suggests that there is no “one size fits all” when it comes to PA postpartum.

Most women did not mention the use of social media or “fit-fluencers” as impacting their PA or overall well-being. Laura, who retained postpartum weight, noted that since she was searching for more content that she related to on body positivity, algorithms ensured that what was showing up in her feed was appropriate to where she was at with her body and fitness. This was parallel to Hannah who had an education in this area and therefore followed social media accounts in her field. However, even following social media accounts that were appropriate resources didn’t eliminate social comparison. Hannah who retained postpartum weight discusses this challenge in following a mother who was a physiotherapist but had a very fit body.

#### Finding relatable PA information can be challenging

This subtheme captures when women shared their perspectives around their challenge of finding PA information that was relatable. Many mothers noted a lack of relatable postpartum imagery and information, emphasizing that they did not feel represented in the resources available to them. Sarah who retained postpartum weight shared, *“a lot of the major advice is geared towards this very specific ideal. . . like women who bounce back quickly and have access to more time or resources than I do. And I think it makes me feel like there’s a gap in representing women of different body types and also the ethnicities and lifestyles.”* She went on to describe how this lack of representation made her feel like she did not belong, and as though she was not doing it right. Ashley (postpartum weight retention) added to this, sharing that the postpartum fitness images she’s encountered are of moms who are already very fit or were clearly personal trainers: *“and it’s like, okay, except that’s not my situation.”* These reflections point to a gap between postpartum exercise messaging and the diverse experiences and bodies of postpartum women.

When asked what kind of imagery they would like to see, Sarah who retained postpartum weight shared *“I would love to see imagery that reflects a more diverse range of postpartum bodies, especially those of black women and women of colour.”* She expressed a desire to see different body shapes and sizes, including those with stretch marks and scars, alongside authentic moments like holding a baby, getting rest, or fitting in a quick stretch. She concluded that *“if the imagery included a wider variety of bodies and experiences, I think I’ll feel more encouraged to take part in physical activities.”* This highlights the need for inclusive PA representation. While some mothers found content they related to, many others struggled to find images and information that demonstrated their lived reality.

#### PA is meant for every-BODY

This subtheme captures the importance noted largely by those who lost postpartum weight that PA is for bodies of all shapes and sizes and not just for those who meet the thin ideal. Comparatively, those who had retained postpartum weight were more likely to engage in the bounce back narrative. However, not everyone who retained postpartum weight felt negatively impacted by comparison and were comfortable following exercises from slimmer moms. For instance, Alyssa (postpartum weight retention) states *“she’s a mom, she’s thinner than me, but it doesn’t make me feel uncomfortable or anything.”* This was even discussed as being a motivating factor in seeing that mothers could have divergent bodies. *“Probably one in three hundred would want to stay fat and all of that. But many people would want to snatch their bodies back”* (Taylor, postpartum weight loss).

Those who retained postpartum weight were more likely to compare to others and have goals that focused on weight loss. Hannah who retained postpartum weight discusses how she would like to see more representation of what postpartum bodies that are strong but body diverse look like doing PA *“not everyone’s going to be having tight skin all over their body, if you’re doing like a core exercise, like bird dog, right, where you’re on all fours and you have to resist gravity, so you’re using your abs, but there’s still all that skin that’s hanging down like you it’s not that you’re doing an exercise wrong.”*

#### PA is a means of controlling body shape and size postpartum

This subtheme captures how PA was often discussed as a means of trying to change one’s body shape and size. While some mothers who lost weight preferred stock images; *“I mean, I’m smaller than the ‘diverse image’, so I guess automatically . . . I’m thankful that I’m smaller that I’m not starting from that baseline. I guess that would be my initial thought. It’s harder for her. I’m thankful that that’s not my reality”* (Kayla, postpartum weight loss) and thought it was a motivating goal, others talked about appreciating the more realistic imagery as it was inclusive of motherhood. However, despite this idealized body neutrality, it was clear that many still focused on the outcome of weight loss in viewing thinner body types postpartum *“every woman is trying to get into shape, is trying to get fit.”* Nicole (postpartum weight retention) reflects needing to engage in exercise to achieve weight loss immediately after delivery where she was heavier than during pregnancy *“So after delivery, I had to do some home exercises to drop down weight, because ideally, I don’t really like being fat, so I had to do some exercise, like walking around and then doing some planks, dancing at home, doing sit ups”*.

## Discussion

Postpartum women may experience challenges to PA engagement and body dissatisfaction. The purpose of this work was to explore postpartum women’s perceptions of their body (body dissatisfaction), PA, and related constructs (PA identity, exercise self-efficacy, self-compassion) and evaluate whether our newly created PA imagery comprised of more “realistic” and diverse bodies influenced these perceptions. Postpartum women in this study reported largely meeting PA guidelines and when exploring quantitative data there was no difference in measures of exercise self-efficacy, PA identity, body dissatisfaction, or self-compassion based on weight status (i.e. those who reported weight retention did not differ from those who reported weight loss postpartum). However, thematic analysis provided insight into potential nuances not captured by these measures and underscored the overall perceptions of PA imagery on body dissatisfaction as well as unique differences between those who did and did not retain weight postpartum.

The finding that postpartum women who lost weight were able to appreciate their body as an impressive feat of motherhood and were able to embrace their bodies and imperfections aligns with previous work (Fern et al., 2012). Novel to this study is that the appreciation of one’s body post birth is more evident amongst those who were able to lose their postpartum weight. Rallis et al. (2007) report that 87.4% of postpartum women wanted a smaller body compared to 75.5% pre-pregnancy and therefore there may be divergent perspectives between those who maintain versus lose their postpartum weight. This may suggest that the bounce back narrative and body appreciation are in conflict and create confusion. As found by DeLuca and Bustad (2017), body dissatisfaction and body appreciation can co-exist postpartum and that this is not a “uni-directional, or fixed experience” (Raspovic et al., 2022).

Most women reported meeting the PA guidelines of 150 minutes of moderate-to-vigorous PA per week in this study sample, which may be due to their higher PA identity than shown in our previous work with postpartum women (Lesser et al., 2023). Although most women postpartum report reduced PA or have not returned to pre-pregnancy PA levels, the positive return is also observed in previous research (Raspovic et al., 2022). Notably, this may also be due to potential risk of selection bias given the participants knew the subject area covered by this research study was related to PA. Our participants largely expressed seeking out PA information from credible sources that were appropriate to postpartum which may have positively impacted their confidence around PA and increased engagement. However, those who retained postpartum weight were more likely to see PA as a means of controlling body shape and size rather than appreciating the many mental and physical health benefits of PA postpartum regardless of weight loss (Davenport et al., 2025). Previous work suggests that those with higher body appreciation expressed a more positive return to postpartum PA (Erbil et al., 2012) than those who purely engage in PA as a means of changing their body shape (Raspovic et al., 2022).

Given the previous findings that postpartum women struggle with PA engagement, and this may be associated with body dissatisfaction and social comparison, we further assessed the views of postpartum women after viewing stock versus realistic imagery of postpartum exercises. We found postpartum women in our study to appreciate more realistic imagery with commentary reflecting a preference for less esthetic images (i.e. messy homes and less tranquil settings). Social media accounts which feature postpartum women have also been found to depict exercise demonstrations in bright locations with no clutter (Jones et al., 2024). Participants who retained postpartum weight were more likely to discuss the size of the body in the image and were more attracted to the stock photo as aligning with their goals rather than what they depicted as their reality as shown in other studies where weight loss is a central focus amongst those who retain postpartum weight (Chen and Chang, 2023). This preference for a slimmer body in exercise images aligns with the discourse that imagery depicted around pregnancy and postpartum representing “impossible standards” (Liechty et al., 2018) yet led to women subconsciously believing this to be the norm (Jones et al., 2024). However, women broadly suggested that they appreciated seeing both forms of imagery, though it did not appear to alter how they felt about their own bodies or their PA engagement suggesting a resiliency to the negative influences of social media if they can embrace their body as being functionally capable after motherhood (Tiggemann and Zaccardo, 2018). However, it is possible that the stock imagery was less impactful than what was shown in exposure to body-focused social media posts postpartum where body dissatisfaction was enhanced (Tang et al., 2022).

Strengths of this study include the mixed methods approach allowing for a deeper understanding of the postpartum experience through both quantitative and qualitative data. In addition, creation of our own “realistic” imagery in comparison to stock imagery is an innovative step in understanding how to best support postpartum women in navigating postpartum PA independent of the socially constructed bounce back narrative. Limitations of the created imagery for this study is the lack of representative racial diversity, while the chosen models were intensity to be racially representative for the location in which the research was taking place (South Asian and European), this did not resonate with the sample which was largely African American. Therefore, we are unable to discuss our imagery as being more racially diverse than the comparative stock imagery. While we intended to assess whether there may have been potential differences between those who retained postpartum weight versus those who did not, we did not ask about whether individuals were struggling with their body weight or body dissatisfaction prior to having children which may have impacted how they felt about imagery and PA engagement postpartum. There were differences between those who retained versus lost postpartum weight in age of their infant and therefore it is unknown if time since birth acted as an additional factor in discussing body dissatisfaction and PA. Lastly, the study team is comprised of women, three of which have children, and while attempts were made to minimize bias, it is recognized that one’s own experiences may impact data interpretation.

Taken together, findings from this study suggest women experience body dissatisfaction and social comparison when/after viewing PA imagery and desire for more “realistic” PA imagery. Despite no quantitative differences in those who did and did not retain weight postpartum, interesting nuances were evident in the qualitative data, which provide possible targets for intervention such as strategies for PA engagement that focus on health over size amongst those who struggle with postpartum weight retention. Findings also underscore the necessity of further efforts to develop and disseminate “realistic” PA imagery to ensure postpartum women know that PA is for every-BODY. Future research should consider whether engagement with “realistic” PA imagery influences PA engagement and body dissatisfaction in this population.

## Supplemental Material

sj-docx-1-hpq-10.1177_13591053261419659 – Supplemental material for MomBod: Exploring body-related perceptions and the influence of physical activity imagery among postpartum women Supplemental material, sj-docx-1-hpq-10.1177_13591053261419659 for MomBod: Exploring body-related perceptions and the influence of physical activity imagery among postpartum women by Iris A. Lesser, Megan Filiatrault, Amanda Wurz and Taniya S. Nagpal in Journal of Health Psychology

sj-docx-2-hpq-10.1177_13591053261419659 – Supplemental material for MomBod: Exploring body-related perceptions and the influence of physical activity imagery among postpartum women Supplemental material, sj-docx-2-hpq-10.1177_13591053261419659 for MomBod: Exploring body-related perceptions and the influence of physical activity imagery among postpartum women by Iris A. Lesser, Megan Filiatrault, Amanda Wurz and Taniya S. Nagpal in Journal of Health Psychology

sj-docx-3-hpq-10.1177_13591053261419659 – Supplemental material for MomBod: Exploring body-related perceptions and the influence of physical activity imagery among postpartum women Supplemental material, sj-docx-3-hpq-10.1177_13591053261419659 for MomBod: Exploring body-related perceptions and the influence of physical activity imagery among postpartum women by Iris A. Lesser, Megan Filiatrault, Amanda Wurz and Taniya S. Nagpal in Journal of Health Psychology

## Data Availability

Data associated with this manuscript is available upon request.

## References

[bibr1-13591053261419659] Baker B Yang I (2018) Social media as social support in pregnancy and the postpartum. Sexual & Reproductive Healthcare 17: 31–34. 10.1016/j.srhc.2018.05.003 30193717

[bibr2-13591053261419659] Braun V Clarke V (2019) Reflecting on reflexive thematic analysis. Qualitation Research in Sport, Exercise and Health 11(4): 589–597.

[bibr3-13591053261419659] Braun V Clarke V (2025) Reporting guidelines for qualitative research: a values-based approach. Qualitative Research in Psychology 22(2): 399–438.

[bibr4-13591053261419659] Brewer B Van Raalte J Linder D (1993) Athletic identity: Hercules’ Muscles or Achilles Heel? International Journal of Sport Psychology 24: 237–254.

[bibr5-13591053261419659] Chen ML Chang SR (2023) The relationship between body dissatisfaction and postpartum depressive symptoms: A cross-sectional study. Journal of Affective Disorders 324: 418–423. 10.1016/j.jad.2022.12.102 36586599

[bibr6-13591053261419659] Chu AHY Padmapriya N Tan SL , et al. (2023) Longitudinal analysis of patterns and correlates of physical activity and sedentary behavior in women from preconception to postpartum: The Singapore preconception study of long-term maternal and child outcomes cohort. Journal of Physical Activity and Health 20: 850–859. 10.1123/jpah.2022-0642 37146982

[bibr7-13591053261419659] Clark A Skouteris H Wertheim EH , et al. (2009a) My baby body: A qualitative insight into women’s body-related experiences and mood during pregnancy and the postpartum. Journal of Reproductive and Infant Psychology 27: 330–345.

[bibr8-13591053261419659] Clark A Skouteris H Wertheim EH , et al. (2009b) The relationship between depression and body dissatisfaction across pregnancy and the postpartum: A prospective study. Journal of Health Psychology 14: 27–35. 10.1177/1359105308097940 19129334

[bibr9-13591053261419659] Davenport MH Meyer S Meah VL , et al. (2020) Moms are not OK: COVID-19 and maternal mental health. Frontiers in Global Women’s Health 1: 1. 10.3389/fgwh.2020.00001 PMC8593957 34816146

[bibr10-13591053261419659] Davenport MH Ruchat SM Jaramillo Garcia A , et al. (2025) 2025 Canadian guideline for physical activity, sedentary behaviour and sleep throughout the first year post partum. British Journal of Sports Medicine 59: 515–526. 10.1136/bjsports-2025-109785 40139673

[bibr11-13591053261419659] DeLuca JR Bustad JJ (2017) ‘My baby and this mom body’: Examining post-partum bodywork through stroller fitness. Qualitative Research in Sport Exercise and Health 9: 133–148.

[bibr12-13591053261419659] Dipietro L Evenson KR Bloodgood B , et al. (2019) Benefits of physical activity during pregnancy and postpartum: An umbrella review. Medicine and Science in Sports and Exercise 51: 1292–1302. 10.1249/MSS.0000000000001941 PMC6527310 31095086

[bibr13-13591053261419659] Erbil N Senkul A Başara GF , et al. (2012) Body image among Turkish women during the first year postpartum. Health Care for Women International 33: 125–137. 10.1080/07399332.2011.603977 22242653

[bibr14-13591053261419659] Evans C Dolan B (1993) Body shape questionnaire: Derivation of shortened “alternate forms”. International Journal of Eating Disorders 13: 315–321. 10.1002/1098-108x(199304)13:3<315::aid-eat2260130310>3.0.co;2-3 8477304

[bibr15-13591053261419659] Fern VA Buckley E Grogan S (2012) Women’s experiences of body image and weight loss after childbirth. British Journal of Midwifery 20: 860–865.

[bibr16-13591053261419659] Gjerdingen D Fontaine P Crow S , et al. (2009) Predictors of mothers’ postpartum body dissatisfaction. Women & Health 49: 491–504. 10.1080/03630240903423998 PMC2796197 20013517

[bibr17-13591053261419659] Hatfield G Lesser I Nienhuis C (2022) Utility of an outdoor group exercise program for improving postpartum mental health. Health and Fitness Journal of Canada 15: 18–30.

[bibr18-13591053261419659] Horton E Everett B Romito M (2025) Inundated with “Bounce Back” Culture: An interpretative phenomenological analysis of postpartum first-time mothers’ body image dissatisfaction and Mental Health Implications. Family Journal 33: 470–478.

[bibr19-13591053261419659] Huang TT Dai FT (2007) Weight retention predictors for Taiwanese women at six-month postpartum. Journal of Nursing Research 15: 11–20. 10.1097/01.jnr.0000387595.94413.90 17370229

[bibr20-13591053261419659] Jones HM Orr J Whelan ME , et al. (2024) An exploration of pregnancy and postpartum content on Instagram: A content analysis of health and exercise focused accounts. Women and Birth 37: 101632. 10.1016/j.wombi.2024.101632 38971136

[bibr21-13591053261419659] Lesser I Wurz A Bean C , et al. (2024) Exploring the feasibility, acceptability, and potential benefits of the mom Movement Intervention (MOMmi). Nursing for Women’s Health 28: 264–276. 10.1016/j.nwh.2024.01.006 38782044

[bibr22-13591053261419659] Lesser IA Turgeon S Nienhuis CP , et al. (2023) Examining the role of physical activity on psychological well-being and mental health postpartum. Women in Sport and Physical Activity Journal 31: 40–49.

[bibr23-13591053261419659] Liechty T Coyne SM Collier KM , et al. (2018) “It’s just not very realistic”: Perceptions of media among pregnant and postpartum women. Health Communication 33: 851–859. 10.1080/10410236.2017.1315680 28467123

[bibr24-13591053261419659] Nagl M Jepsen L Linde K , et al. (2021) Social media use and postpartum body image dissatisfaction: The role of appearance-related social comparisons and thin-ideal internalization. Midwifery 100: 103038. 10.1016/j.midw.2021.103038 34051430

[bibr25-13591053261419659] Neff K (2003) Self-compassion: An alternative conceptualization of a healthy attitude toward oneself. Self and Identity 2: 85–101.

[bibr26-13591053261419659] Neupert SD Lachman ME Whitbourne SB (2009) Exercise self-efficacy and control beliefs: Effects on exercise behavior after an exercise intervention for older adults. Journal of Aging and Physical Activity 17(1): 1–16. 10.1123/japa.17.1.1 PMC3740728 19299835

[bibr27-13591053261419659] Peralta LR Cotton WG Dudley DA , et al. (2021) Group-based physical activity interventions for postpartum women with children aged 0–5 years old: A systematic review of randomized controlled trials. BMC Women’s Health 21: 435. 10.1186/s12905-021-01581-1 PMC8714424 34963456

[bibr28-13591053261419659] Raes F Pommier E Neff KD , et al. (2011) Construction and factorial validation of a short form of the Self-Compassion Scale. Clinical Psychology & Psychotherapy 18: 250–255. 10.1002/cpp.702 21584907

[bibr29-13591053261419659] Rallis S Skouteris H Wertheim EH , et al. (2007) Predictors of body image during the first year postpartum: A prospective study. Women & Health 45: 87–104. 10.1300/J013v45n01_06 17613464

[bibr30-13591053261419659] Raspovic AM Hart LM Zali Y , et al. (2022) Body image profiles and exercise behaviours in early motherhood. A latent profile analysis. Journal of Health Psychology 27: 2056–2067. 10.1177/13591053211019114 34030494

[bibr31-13591053261419659] Ritondo T Bean C Lesser I (2024) ‘I didn’t know who to ask about how it should feel’: Postpartum women navigating the return to physically active leisure. Leisure/Loisir 48: 475–504.

[bibr32-13591053261419659] Rosenbaum DL Gillen MM Hutson DJ (2024) The relationship between social media use and pregnancy-related body image. Women’s Health Epub ahead of print 19 December 2024. DOI: 10.1177/17455057241309496. PMC11656518 39698958

[bibr33-13591053261419659] Saligheh M McNamara B Rooney R (2016) Perceived barriers and enablers of physical activity in postpartum women: A qualitative approach. BMC Pregnancy and Childbirth 16: 131. 10.1186/s12884-016-0908-x PMC4890285 27256279

[bibr34-13591053261419659] Spinoni M Singh Solorzano C Grano C (2023) A prospective study on body image disturbances during pregnancy and postpartum: The role of cognitive reappraisal. Frontiers in Psychology Epub ahead of print 9 August 2023. 14. DOI: 10.3389/fpsyg.2023.1200819. PMC10444978 37621944

[bibr35-13591053261419659] Tang L Tiggemann M Haines J (2022) #Fitmom: An experimental investigation of the effect of social media on body dissatisfaction and eating and physical activity intentions, attitudes, and behaviours among postpartum mothers. BMC Pregnancy and Childbirth 22: 766. 10.1186/s12884-022-05089-w PMC9555257 36224523

[bibr36-13591053261419659] Thompson KA Bardone-Cone AM (2022) Social comparison, disordered eating, and body dissatisfaction among postpartum women. Body Image 42: 401–412. 10.1016/j.bodyim.2022.07.011 35930875

[bibr37-13591053261419659] Tiggemann M Zaccardo M (2018) ‘Strong is the new skinny’: A content analysis of #fitspiration images on Instagram. Journal of Health Psychology 23: 1003–1011. 10.1177/1359105316639436 27611630

[bibr38-13591053261419659] Tornero-Quiñones I Rendón Gallego M Molina-López J , et al. (2023) La relación de la actividad física sobre la imagen corporal en mujeres postparto. Psychology, Society and Education 15: 37–44.

[bibr39-13591053261419659] Vanderkruik R Ellison K Kanamori M , et al. (2022) Body dissatisfaction and disordered eating in the perinatal period: an underrecognized high-risk timeframe and the opportunity to intervene. Archives of Women’s Mental Health 25: 739–751. 10.1007/s00737-022-01236-6 35524142

[bibr40-13591053261419659] Wenhold H Couture Bue AC Kirkpatrick CE (2025) #Bopo or bounce back?: Investigating the impact of social media videos on postpartum mothers. Health Communication 40: 2674–2685. 10.1080/10410236.2025.2475856 40114468

[bibr41-13591053261419659] Yelland J Sutherland G Brown SJ (2010) Postpartum anxiety, depression and social health: Findings from a population-based survey of Australian women. BMC Public Health 10: 771. 10.1186/1471-2458-10-771 PMC3022850 21167078

