# Supplementary material for: MomBod: Exploring body-related perceptions and the influence of physical activity imagery among postpartum women
Source: J Health Psychol. 2026 Feb 22;31(12):4785–99. doi: 10.1177/13591053261419659 (PMC13612885; doi:10.1177/13591053261419659)
Supplement: Supplementary file 1 [file sj-docx-1-hpq-10.1177_13591053261419659.docx]

| **Theme label and description** | **Subtheme label and description** | **Sample quotations** |
| --- | --- | --- |
| **Perception around body image and size postpartum** |  | “Not great. You sort of look at yourself and it's like, who is that person?” (Laura, PWR)  “I don't really like the way I am right now, but at the same time, I'm not dwelling on it too much. I guess I'm kind of just like, it is what it is. I just made a person” (Ashley, PWR)  “I have mixed feelings about my body right now. On one hand, I'll say I'm amazed at what it has done to me in body. Growing and giving back to my baby feels like, I'll say that such a powerful thing. But, on the other hand, it's hard to notice the changes, like, I said the extra weight, the stretch marks, and how things feel different overall. I'm sort of kind of trying to be kind to myself and focus on what my body has achieved rather than how it looks, and I'd say, some days it's easier than others, but I'm more accepting and appreciating this new version of me.” (Sarah, PWR) |
|  | 1. **The body as a battleground** | “I think my body's amazing for what it did for getting pregnant and having babies. But it is hard because your body changes so much.” (Alyssa, PWR)  “More of a mental thing than a physical thing that you don't feel like your body belongs to you anymore” (Leah, PWL)  “I'm definitely trying to change the narrative, but it's so hard. Like, I can look in the mirror and be like, I'm happy with who I am. I'm like, this is okay. And then I look at pictures, and particularly with looking at pictures of myself, yeah, that is the hardest part.” (Hannah, PWR)  “I always think about when I'm done breastfeeding and stuff like that, then I'll really get my body back, because it still serves a purpose for the kids.” (Alyssa, PWR)  “It's not the same anymore, and I just don't feel confident. I don't feel like I look beautiful. And I just keep questioning myself every day, like, why did I go into this? I didn't know having kids would be so hard on the body. And I'm trying my best to stay positive in the back of my head. But in the forefront of my brain, there is like so many questions when I look in the mirror.” (Emily, PWR)  “My body is not the same anymore. So I kind of struggle every day to accept myself, but when I see my baby, I think it's all worth it. I think I'll do it again if I get a chance. But you know, it's kind of been a roller coaster of emotions to me.” (Julie, PWR)  “It hasn't necessarily been what I look like in the mirror, but it's been like when I notice my clothes don't fit me the way that they used to or because I had a C-section like certain things still irritate my scar and so then it's not the clothes don't fit but they don't feel good on my body” (Elizabeth, PWL)  “I think I look really good for six months postpartum, but I am not where I want to be. So yeah, it depends on the day. Some days I'm like, Yeah, I look great. I should be really proud of myself. And other days, I get into a little bit of a darker hole.” (Kayla, PWL) |
|  | 2. **Embracing the “mom-bod”** | “I'm not really stressing about weight because I'm currently breastfeeding, so I don't feel the necessary pressure to bounce back to my normal body. And I've also sort of gained some scars and so I'm embracing them and I'm really hoping that they stay on. And I'm loving my body right now.” (Kayla, PWL)  “Postpartum, I feel like I'm a little bit more accepting. I give myself more grace. I don't look at food as good versus bad, but rather than healthy versus you know, I can have this treat today and it's not going to kill me. So I'm definitely more lenient” (Kayla, PWL)  “Like, I feel stronger. I'm also just in awe. Like my skin is different; it's a different texture. It feels different than it did before, like it's very loose, you know? So, it looks different, but I'm just really proud of it and I actually feel really, like this is probably, like the best body image I've had in my last like 10 15 years, I would say” (Dana, PWL)  “… I feel like, if anything, I just have more appreciation for my body because of the incredible miracle that it produced, which is actually just blows my mind, still to this day, that I look at my baby and I'm like, I can't believe my body grew that” (Leah, PWL)  “I'm filled with joy just to look the way I look and to be who I am, to be a mother to someone. Feels great about my body.” (Jasmine, PWL)  “As much as I would like to get back in shape, you know, get back like my old body, I also try to embrace the new me.” (Lisa, PWR) |
| **PA imagery preferences and needs** | 1. **Retention of postpartum weight impacts how women view exercise imagery** | “It would be the mix of in the house, outside, in a hotel gym, in an unaesthetic basement gym… where they're doing it, and without the laundry being clean first, you know” (Dana, postpartum weight loss).  “Women may look like if they rolled out of bed and wanted to squeeze a 20-minute home workout in.” (Elizabeth, PWL)  “Babies are different than others, so they might be dealing with a very colicky, crying baby or something, and that could affect them in a certain way”. (Mary, PWL)  “It would be nice to see imagery of moms trying to do a class and how like getting interrupted because their kid is doing something like, how do they recover when everybody's not like, sitting perfectly in a row and the babies are all just happy and sitting there. (Laura, PWR)  “My muscles don't do what they used to, not as strong as I was before. So, it's encouraging seeing the ‘diverse image’ exercises, because they're all like getting into it where they're at. You know what I mean, whereas the other photos obviously like I said look a bit more staged and glamorized and not as real and relatable.” (Mary, PWR)  “Mom one did more yoga based simpler movements that are designed by a pelvic health physio for postpartum, and mom two is doing exercises that are a little bit too advanced for the newly postpartum woman” (Anna, PWL).  “it's so realistic. It's how everyone looks after pregnancy. I mean, ‘stock imagery’ really looks like they just picked up a model who hasn't been pregnant before and had to do a shoot or something…. definitely feel more motivated to work out because she's doing it.” (Julie, PWR).  “People who actually see like the plus size mom may probably think she needs lots of work, and some people might think, because the other person is slim, that she doesn't need lots of work.” (Lisa, PWL)  “I still kind of wish I looked more like ‘stock imagery'”, however, she also states: “it's reassuring to know that there's that I'm definitely not the only mother that is going through this stage of life that doesn't look like the Pilates mom in there...” (Hannah, PWR)  “Like, plus size, a little bit of plus size, then also blacks” (Nicole, PWR) |
|  | 2. **Social comparison hinders confidence and self-acceptance in motherhood** | “I have mom friends that didn't gain much weight at all in pregnancy, and they look very similar like postpartum as they did pre pregnancy. And that's when comparisons start to happen, and it starts to get a little bit more negative self-talk going on there.” (Hannah, PWR)  “I felt so insecure, you know, most of the time, because I know I've seen so many women have children, but they bounced back to shape so easily. So I kind of wonder, why is it so hard for me? Why can’t I achieve that?” (Julie, PWR)  “It was kinda funny because I was telling you, I was like, yeah, that's a model, and that's just somebody's baby that was brought there. But I think when I go online and I don't see that comparison, I'm not thinking about it” (Ashley, PWR)  “When there is a comparison, it's like, oh, that's a model, and then there's the real mom. But when there's no comparison and you're just going through a series of models at god knows what hour, your brain just starts going...” (Ashley, PWR)  “Why can't I get a tummy tuck? You know what I mean? Like is everybody spending $30,000 on a tummy tuck, and that's the way that we go to the gym and these crop top matching sets or whatever the thing is. So, I think it made me have these questions of, like, why not me?” (Laura, PWR)  “I feel a bit discouraged at times especially when I don't see women who look like me or have similar experiences and it made me feel like my journey isn't as valid or that I'm behind in some way.” (Sarah, PWR)  “… Like why can't I be like that, you know? Or like, how did she get to be there? And like, that's what the negative thinking starts. Is it a bit discouraging? Yes, but, like, also, I don't know, I can look past it I guess…I have mom friends that didn't gain much weight at all in pregnancy, and they look very similar like postpartum as they did pre pregnancy. And that's when comparisons start to happen, and it starts to get a little bit more negative self-talk going on there” (Hannah, PWR)  “Honestly, like, I still kind of wish I looked more like mom number two (stock image). Um, so that's, I guess, my honest initial reaction” (Hannah, PWR)  “She's all about ab rehab, which is great, like, we know how important that is, but she has a major six pack, she is the leanest woman I think I've ever seen, and she has two babies, and that's sometimes hard to see. Like why can't I be like that, you know? Or like, how did she get to be there? And like, that's what the negative thinking starts. Is it a bit discouraging? Yes, but, like, also, I don't know, I can look past it I guess.” (Hannah, PWR) |
|  | 3. **Social comparison supports common humanity in motherhood** | “I wouldn't say I want them to be different imagery because like everybody's body is different and everybody's body is going to adapt differently to the different imagery. Once a person can see an image that they think will work for them, then I think that would be the best thing because you cannot replicate somebody else's image. You just have to be true to yourself and love yourself, even though it's hard and sometimes I myself, I feel like I don't love myself.” (Emily, PWR)  “There was one girl, I thought, she put a set, like one of those little towers of barbells where it's like, three, five, ten, or whatever. She put it in her washroom so that when she's brushing her teeth or sitting on the toilet, she could do like, 10 reps of a bicep curl. That's like brilliant, that's like, what a win looks like.” (Laura, PWR)  “I'd like to say I'd like to see a bit of struggling, because those activities are really not so easy to perform. So, I like to see a bit of struggle, to show that, yeah, people are struggling to do this and not just struggling alone.” (Julie, PWR)  “We've got different body shapes. We've got different body sizes. We've got people with different lived experiences. I enjoy reading from the lived experiences of pregnant moms and women we just give birth, and this has kind of prepared me for this phase, for this journey knowing it's not always going to be perfect.” (Taylor, PWR)  I've started following this girl. Her kid is like two days older than my kid. We look the same. We have the same body type; we have the same postpartum story. She posts a lot about her struggles, and her struggles are the same as mine, and she's like listen, I haven't lost any weight” (Laura, PWR) |
| **Sources of PA information** |  | “And I've been doing some walking exercises, a little bit because I saw that generally working out is good for you, not just for your physical body, but also for your mental health. I found that information helpful.” (Jasmine, PWL)  “Presented by other mothers, people who have my experience. I feel like that will be more real.” (Lisa, PWR)  “Mostly focused around what can or can I not do based on a C-section.” (Elizabeth, PWL)  “Few YouTube channels that were “postpartum yoga, postpartum diaphragmatic breathing, postpartum stretch.” And, you know, so I would say on YouTube and Instagram there was people that I trusted.” (Dana, PWL)  “So, before I had kids, I would go to the gym all the time, and then I had kids, and I'm like, Well, I'm never gonna leave my house, so I brought the gym to me. I got a lot of information from social media and just kind of diving into athletes that I look up to, and kind of educating myself on nutrition and that kind of stuff over the years.” (Kayla, PWL)  “I try to find professionals. And then I go to a pelvic floor physio as well.” (Madison, PWR) |
|  | 1. **Finding relatable PA information can be challenging** | “I sort of felt represented, but not entirely. I'll say a lot of the major and advice is geared towards this very specific ideal. I'll say you're, like, women who bounce back quickly and have access to more time or resources than I do. And I think it makes me feel like there's a gap in representing women of different body types and also the ethnicities and lifestyles” (Sarah, PWR)  “So, the stuff I've looked at never exactly represents me per se, but, I mean, it's sometimes motivating anyways to see bodies in different ways” (Alyssa, PWR)  “No. I feel like the pictures are all either AI pictures of someone who's already super fit or some mom who's clearly a personal trainer. And she's like, this is my job, therefore, I do this twenty-four seven. And it's like, okay, except that's not my situation.” (Ashley, PWR)  “I would love to see imagery that reflects more diverse range of postpartum bodies, especially those of black women and other women of color. I think it would be great to see a variety of body shapes and size and some with stretch marks, some with scars, some embracing the changes with confidence. And imagery that shows real moments of postpartum life like holding a baby, getting rest, or I'll say fitting in a quick stretch. I think it will feel more relatable and authentic.” (Sarah, PWR)  “It's a bunch of exercises that cater to people who are struggling with obesity or persons who had a c section and just don't know how to do exercises because they're afraid of how they are going to lift something if they had a cesarian procedure. So that doesn't really cater to me much” (Emily, PWR)  “Most of the activities that they recommend are a little bit hard to perform. So I begin to wonder other women don't go through this or something I don't know” (Julie, PWR)  “Some of them are all advanced and higher more complicated poses, that would affect my confidence. In doing it because I'm like, oh, I can't actually even lift my leg that high or balance.” (Madison, PWR)  “I feel a bit discouraged at times especially when I don't see women who look like me or have similar experiences and it made me feel like my journey isn't as valid or that I'm behind in some way.” (Sarah, PWR) |
|  | 2. **Physical activity is for every-BODY** | “she’s a mom, she’s thinner than me, but it doesn’t make me feel uncomfortable or anything.” (Alyssa, PWR)  “I don't really think it differs because every woman is trying to get into shape, is trying to get fit. Probably one in three hundred would want to stay fat and all of that. But many people would want to snatch their bodies back. They would want to tuck their tummies in” (Taylor, PWR)  “I think everybody looks different and starts from different points, and I'm a big believer that if you see somebody exercising, I really don't care what they look like. I'm like, good for them if they're bigger, and it's not an easy task to do, and everyone needs to start somewhere. And if they're fit, they've probably been doing it for a long time, and that takes discipline. So, I usually view it from that frame of lens. If you're exercising, you're doing what you need to do.” (Kayla, PWL)  “It has made me feel like there's a space for me no matter where I am in my postpartum journey or how my body looks and right now that I've seen only I'll say, those different types of body postpartum fitness images, it has made me feel like, I don't I don't belong or that I'm not doing it right maybe. But I think if the imagery included a wider variety of bodies and experience it is, I think I'll feel more encouraged to take part in the physical activities.” (Sarah, PWR)  “Like, she’s super fit, but she still has mom bod and whatever. And it's fine. Like it's positive.” (Alyssa, PWR)  “not everyone's going to be having tight skin all over their body, if you're doing like a core exercise, like bird dog, right, where you're on all fours and you have to resist gravity, so you're using your abs, but there's still all that skin that's hanging down like it's not that you're doing an exercise wrong.” (Hannah, PWR) |
|  | 3. **Physical activity is a means of controlling my body shape and size** | “I mean, I'm smaller than the ‘diverse image’, so I guess automatically … I'm thankful that I'm smaller that I'm not starting from that baseline. I guess that would be my initial thought. It's harder for her. I'm thankful that that's not my reality” (Kayla, PWL)  “But at the same time, there is the part of me that knows the realistic of it all. So first thought is […] you. Excuse my language. Second thought is, wait you gotta put in the work.” (Madison, PWR)  “People who actually see like the plus size mom may probably think she needs lots of work, and some people might think, because the other person is slim, that she doesn't need lots of work.” (Lisa, PWR)  “What's the point of all this activity, if you still end up looking like you just had a baby? And I know, like, not all of your thoughts or thoughts you should listen to.” (Laura, PWR)  “I'm not treating my body the way it's supposed to be treated because sometimes I'll start a diet and then I'll stop. Sometimes I want to exercise and then I just feel overwhelmed or lazy or just don't have the energy to do it.” (Emily, PWR)  “So, after delivery, I had to do some home exercises to drop down weight, because ideally, I don't really like being fat, so I had to do some exercise, like walking around and then doing some planks, dancing at home, doing sit ups” (Nicole, PWR) |
