# Supplementary material for: MomBod: Exploring body-related perceptions and the influence of physical activity imagery among postpartum women
Source: J Health Psychol. 2026 Feb 22;31(12):4785–99. doi: 10.1177/13591053261419659 (PMC13612885; doi:10.1177/13591053261419659)
Supplement: Supplementary file 2 [file sj-docx-2-hpq-10.1177_13591053261419659.docx]

**Physical activity engagement, body image acceptance and postpartum physical activity imagery**

**Semi-Structured Interview Guide**

Hi, thank you for taking the time to be interviewed today. As you may recall, the purpose of this interview is to understand how you felt about physical activity postpartum and whether images and video footage of real postpartum moms with diverse bodies has an impact on how you feel about physical activity.

I have some information that I am going to share with you now just so you are clear as to what your participation involves. Feel free to interrupt me at anytime if you need or want me to slow down or repeat anything.

All ethics procedures and assurances remain the same for this component of the study. You previously read the consent form when you agreed to participate in the online survey, I can email you another copy of the consent form if you would like? [pause, wait for response and send consent form and/or continue]. As a reminder, your responses and identity will be kept anonymous. As a member of the research team, I will know your identity, but I will assign a code to your name so that no one else from the research team can identify what you will share with me in the interview today.

Your participation is voluntary, and you can withdraw at any time and without any prejudice to you up to one month after completion of the interview. There are no right or wrong answers. Your perspective will help us understand more about the experiences of moms moving more, and what may be useful for future programs.

Of note, some of these questions might sound repetitive, but I will ask them anyway. Know that you can always skip a question if it makes you feel uncomfortable.

This interview will be audio recorded and transcribed verbatim by the research team. Transcribed files will only be used for the purposes of data analysis by the research team. The audio recording will be stored in on a password protected laptop. Do you have any questions related to this interview? [if yes, interviewer to answer/address; if no, continue to next question.] Do you agree to participate in the interview? [if yes, continue; if no, terminate interview after asking if they have a reason and thanking them for their time].

1- Can you tell me about your postpartum journey so far? How old is your child now?

2- How do you feel about your body since having your (most recent) child?

PROBES: How do you feel about your size? How do you feel about your shape? How has motherhood impacted your attitude towards your body?

3- Are you generally happy with your body? Your body image? Why do you say that?

PROBE: What contributes to how you view yourself?

4- Have you looked for postpartum physical activity information? If yes, where do you typically go for information about postpartum physical activity? If no, why not?

PROBES: Why was this your source of information? Did you feel represented in the physical activity information you accessed?

5- Have you used social media to access physical activity information? If yes, can you tell me about how you feel about yourself and your body after accessing postpartum physical activity information? If no, why not?

PROBES: How did this further impact your confidence to be active postpartum if at all?

6- Whether you have accessed physical activity information, we are curious what type of imagery would you like to see depicting physical activity postpartum? What type of imagery do you think other women would like to see?

PROBE: Why do you say that? Do you think what you would like to see and what you think others would like to see differ, why do you think that is?

**Participants view images and videos with diverse bodies.**

7- What is your confidence for physical activity? How do you feel about physical activity? Why do you think that is?

8- How do you feel about your body? Why do you think that is?

**Participants view stock photos and videos.**

9- What is your confidence for physical activity? How do you feel about physical activity? Why do you think that is?

10- How do you feel about your body? Why do you think that is?

11. Now that you have seen both sets, which did you prefer? Why?

12- Do you have any recommendations about physical activity images to promote physical activity postpartum?

13-Do you have anything else that you would like to share before we conclude this interview?
