# Supplementary material for: MomBod: Exploring body-related perceptions and the influence of physical activity imagery among postpartum women
Source: J Health Psychol. 2026 Feb 22;31(12):4785–99. doi: 10.1177/13591053261419659 (PMC13612885; doi:10.1177/13591053261419659)
Supplement: Supplementary file 3 [file sj-docx-3-hpq-10.1177_13591053261419659.docx]

Postpartum Imagery

Diaphragmatic Breathing

Bird Dog

Bear Plank

Abdominal Oblique Cross Crawl

Abdominal Oblique Cross Crawl

Cat Cow

Glute Bridge

Core Stability March

Toe Taps

Kneeling Squat

Squat

Child’s Pose

Down Dog

Upward Facing Dog
